# Comparison of Serological Methods for the Diagnosis of Toxoplasmosis in Pregnant Women

**DOI:** 10.3390/pathogens15040363

**Published:** 2026-03-29

**Authors:** Nássarah Jabur Lot Rodrigues, Danilo Alves de França, Benedito Donizete Menozzi, Aristeu Vieira da Silva, Joelcio Francisco Abbade, Helio Langoni

**Affiliations:** 1 Department of Animal Production and Preventive Veterinary Medicine, São Paulo State University, Botucatu 18618-686, SP, Brazil; nassarah.lot@unesp.br (N.J.L.R.); benedito.menozzi@unesp.br (B.D.M.); 2 Department of Preventive Veterinary Medicine and Animal Health, University of São Paulo, São Paulo 05508-030, SP, Brazil; danilo.mv.sp@gmail.com; 3 Department of Biological Sciences, State University of Feira de Santana, Feira de Santana 44036-900, BA, Brazil; aristeuvsilva@uesf.br; 4 Department of Gynecology and Obstetrics, Botucatu Medical School, São Paulo State University, Botucatu 18618-686, SP, Brazil; joelcio.f.abbade@unesp.br

**Keywords:** *Toxoplasma gondii*, pregnancy, prenatal, serology, public health

## Abstract

Comparative evaluations of different serological methods for the diagnosis of toxoplasmosis in pregnant women remain limited, and the performance of indirect immunofluorescence assay (IFA), modified agglutination test (MAT), and chemiluminescent microparticle immunoassay (CMIA) has not been previously assessed simultaneously in this population. This study aimed to compare the performance of these three serological methods for the detection of *Toxoplasma gondii* antibodies in pregnant women. A total of 469 serum samples were collected from pregnant women receiving prenatal care through the Brazilian public healthcare system. Samples were tested using IFA, MAT, and CMIA for the detection of IgG and IgM antibodies. Statistical analyses included McNemar’s χ^2^ test, Kappa agreement, and Pearson and Spearman correlation coefficients. IFA and MAT showed higher IgG seropositivity rates (53.1% and 51.2%, respectively) compared to CMIA (46.7% and 48.6%). Agreement between CMIA and IFA was moderate for IgG (Kappa = 0.51) and very strong for IgM (Kappa = 0.89). Pearson’s correlation for IgG between CMIA and IFA was moderate (r = 0.678), while Spearman’s correlation for IgM was weak. IFA and MAT demonstrated greater sensitivity for IgG detection than CMIA, while CMIA and IFA performed similarly for IgM. Conventional methods may complement automated systems to improve diagnostic accuracy in prenatal screening.

## 1. Introduction

Toxoplasmosis, caused by the protozoan *Toxoplasma gondii*, is a widely distributed zoonotic infection with significant public health implications, particularly among pregnant women and immunocompromised individuals from vulnerable populations [1]. Although most infections are asymptomatic or present with mild symptoms, primary maternal infection during pregnancy can lead to congenital toxoplasmosis, carrying the risk of miscarriage, fetal malformations, or severe neurological sequelae in the newborn [2]. Therefore, early and accurate diagnosis of infection in pregnant women is essential to ensure proper follow-up and reduce the risk of vertical transmission [3].

The Ministry of Health in Brazil recommends serological testing for toxoplasmosis during the first prenatal visit. This measure enables the identification of immune and susceptible pregnant women, those with acute infection, and those at risk of vertical transmission. These guidelines are based on the detection of anti-*T. gondii* IgG and IgM antibodies. Although no specific assay is mandatory, chemiluminescent microparticle immunoassay (CMIA) is recommended and widely used in routine prenatal screening in the Brazilian public healthcare system and in private laboratories [4,5,6,7].

However, the performance of available techniques can vary significantly due to differences in sensitivity, specificity, antigenic composition, and technical requirements. These variations may compromise clinical interpretation, particularly regarding the distinction between acute and past infections, a differentiation that is critical for therapeutic decision-making during pregnancy [8].

The ELISA, widely used in serological screening, offers good sensitivity and specificity. However, its large-scale implementation may be limited in developing countries due to the need for standardized commercial kits, specialized equipment, imported reagents, and adequate laboratory infrastructure [9]. These limitations directly affect the feasibility of incorporating ELISA into routine diagnostics and epidemiological studies. In resource-limited settings, the selection of serological tests must balance diagnostic accuracy, cost, and operational feasibility. Automated methods such as chemiluminescent microparticle immunoassay (CMIA) provide high sensitivity and speed and are an excellent alternative when available [10]. Conventional methods, such as the indirect immunofluorescence assay (IFA) and the modified agglutination test (MAT), are frequently used due to their affordability and simplicity [11].

Comparative studies between IFA and ELISA, in both humans and animals, have shown good agreement in the detection of *T. gondii* antibodies, with variations attributed to kit sensitivity, stage of infection, and the methodology used, highlighting their value as viable alternatives for diagnosis [12]. However, few studies have compared the diagnostic performance of IFA, MAT, and CMIA in human populations, and to the best of our knowledge, no study has simultaneously evaluated these three methods in pregnant women. This study aimed to compare the performance of IFA, MAT, and CMIA in detecting anti-*T. gondii* antibodies in pregnant women receiving care through Brazil’s public healthcare system at a public hospital that serves as a reference center for prenatal screening.

## 2. Materials and Methods

### 2.1. Study Area and Samples

This study included 469 serum samples from pregnant women who received prenatal care between 2020 and 2021 at the Clinical Hospital of Botucatu and basic health units in Botucatu and neighboring cities in the Cuesta Pole region of São Paulo state, Brazil. The region encompasses both urban and rural areas, providing a socioeconomically diverse population. All participants underwent routine prenatal screening, during which blood samples were collected, during the first trimester of pregnancy. Women aged 18 and over were included, regardless of underlying conditions or co-infections. After routine diagnostic testing, 100 µL serum aliquots were transferred to labeled tubes and stored at −80 °C for subsequent analysis at the laboratory. Due to sample volume availability, different subsets of samples were used in each serological comparison. The exclusion of samples was based solely on serum volume availability and was not related to the serological results. For IgG analysis, 469 samples were tested using both IFA and CMIA, and 416 samples were tested using CMIA and MAT. For IgM detection, 428 samples were tested using both IFA and CMIA.

### 2.2. Indirect Immunofluorescence Assay (IFA)

The detection of IgG and IgM antibodies against *T. gondii* was performed as described by Pinto et al. [13]. Fluorescence was evaluated under a fluorescence microscope, and samples showing specific cytoplasmic fluorescence in tachyzoites were considered positive. Serum samples were serially diluted, and an IgG titer of 1:16 or higher was considered reactive according to the standard protocol routinely used in the laboratory and previously described [13]. This cutoff has been widely used in serological studies for the detection of anti-*T. gondii* antibodies and was not specifically optimized for pregnant women.

### 2.3. Modified Agglutination Test (MAT)

The detection of IgG antibodies against *T. gondii* was performed as described by Desmonts and Remington [14]. Formalin-fixed tachyzoites of the RH strain of *T. gondii*, maintained in our laboratory and propagated according to standard protocols, were used as antigens, prepared according to the original method described by the authors [14]. Diluted serum samples were added to U-bottom microplates. The presence of a fine agglutination mesh covering the bottom of the well was interpreted as a positive reaction, whereas the formation of a compact button at the bottom indicated a negative result. The addition of 2-mercaptoethanol was used to inactivate IgM antibodies, allowing the specific detection of IgG antibodies. Samples with titers of ≥1:25 were considered positive, following the criteria established in the original method [14]. As with the IFA, this cutoff represents a standard serological criterion and was not specifically adjusted for pregnant women.

### 2.4. Chemiluminescent Microparticle Immunoassay (CMIA)

The detection of IgG and IgM antibodies against *T. gondii* was performed as described by Capobiango et al. [15], using the Architect system with the Architect Toxo IgG and Toxo IgM commercial kits (Abbott Laboratories, Wiesbaden, Germany). The assay employs recombinant antigens p30 (SAG1) and p35 (GRA8) and generates quantitative results. According to the manufacturer’s instructions, IgG results were interpreted as negative (<1.6 AU/mL), equivocal (1.6–3.0 AU/mL), or positive (≥3.0 AU/mL), while IgM results were interpreted as negative (index < 0.50), equivocal (0.50–0.59), or positive (≥0.60). For the purposes of comparison with the other serological methods, results were categorized as reactive or non-reactive according to these criteria.

### 2.5. Data Analysis

Contingency tables and descriptive statistics were generated using EpiInfo™ version 7.2 (Centers for Disease Control and Prevention, Atlanta, GA, USA). The independence between test results was evaluated using McNemar’s χ^2^ test. Measures of association were calculated using the DagStat spreadsheet [16], with a significance level set at α = 0.05.

## 3. Results

Although 469 serum samples were collected, subsets of samples were used in each serological comparison due to availability of sample volume. For IgG analysis, 469 samples were tested using both IFA and CMIA, 416 samples were evaluated with CMIA and MAT, and 428 samples were assessed for IgM using both IFA and CMIA.

Table 1 shows the agreement between CMIA and IFA for IgG detection. The McNemar’s test indicated a statistically significant difference between the two methods (χ^2^ = 115.03; *p* < 0.0001). The agreement rate was moderate (Kappa = 0.51; 95% CI: 0.44–0.57), with positive and negative concordances of 69% and 79%, respectively.

Table 2 presents the comparison between CMIA and MAT. Similarly, McNemar’s test showed significant differences (χ^2^ = 92.04; *p* < 0.0001), with a slightly higher agreement (Kappa = 0.54; 95% CI: 0.47–0.61). Positive concordance was 71%, and negative concordance was 81%.

For IgM detection, the comparison between CMIA and IFA is shown in Table 3. No statistically significant difference was found (McNemar’s χ^2^ = 0.40; *p* = 0.5241), and the agreement was very strong (Kappa = 0.89; 95% CI: 0.82–0.96). The positive and negative concordance rates were 90% and 99%, respectively.

**Figure 1:**
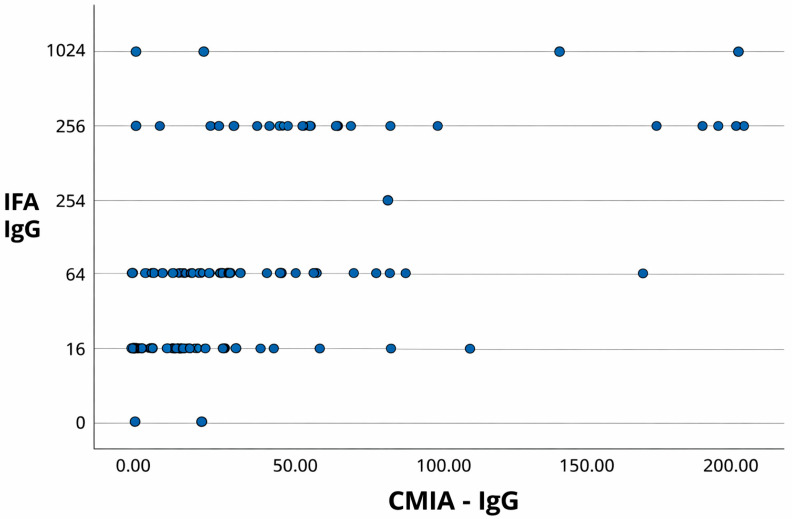
Scatterplot comparing CMIA and IFA results for the detection of IgG antibodies against *T. gondii* in serum samples from pregnant women. CMIA values are expressed as index values, and IFA results are presented as antibody titers (reciprocal serum dilutions). A moderate positive correlation was observed (Pearson’s r = 0.678; 95% CI: 0.628–0.723; *p* < 0.001).

**Figure 2:**
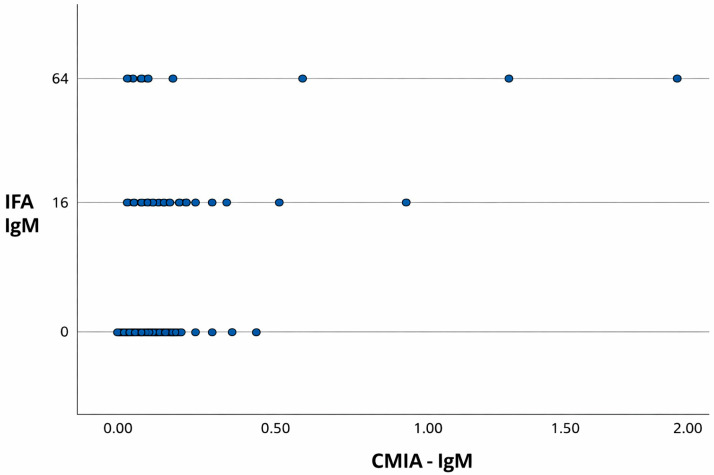
Scatterplot comparing CMIA and IFA results for the detection of IgM antibodies against *T. gondii* in serum samples from pregnant women. CMIA values are expressed as index values and IFA results are presented as antibody titers (reciprocal serum dilutions).

In addition to the contingency analyses, scatterplots comparing CMIA and IFA results for IgG and IgM detection are presented in Figure 1 and Figure 2, respectively.

A moderate positive correlation was observed for IgG (Pearson’s r = 0.678; 95% CI: 0.628–0.723; *p* < 0.001), supporting the concordance between the two methods. In contrast, a weak Spearman correlation was observed for IgM, indicating limited agreement between CMIA and IFA for detecting recent infections.

## 4. Discussion

Despite the importance of using serodiagnostic methods in seroepidemiological surveys of *T. gondii*, comparing results is difficult because of differences in techniques, cut-off points, reagents, equipment, and sampling. Thus, comparisons are more valuable when made in the same study. Since no independent reference method was available for all samples, the present study focused on agreement analyses rather than on estimates of sensitivity and specificity.

In samples with discordant results, discrepancies in IgG detection were more frequently observed when comparing CMIA with IFA and MAT. Specifically, 118 samples were positive by IFA but negative by CMIA, whereas only one sample showed the opposite pattern. Similarly, 95 samples were positive by MAT but negative by CMIA, while only one sample was positive by CMIA but negative by MAT. These findings suggest a potential lower sensitivity of the automated method, particularly in low-titer samples, which is supported by the moderate Kappa agreement and the statistically significant differences observed in McNemar’s tests. Discordant results may also be related to low antibody titers, which can affect the detection capacity of different assays. However, additional confirmatory testing of discordant samples was not performed in the present study.

For IgM detection, however, the agreement between CMIA and IFA was very strong (Kappa = 0.89), with positive and negative concordance rates of 90% and 99%, respectively, and no statistically significant difference according to McNemar’s test (*p* = 0.5241). These findings indicate that both methods can be reliably used for identifying acute infections.

Although enzyme-linked immunosorbent assays (ELISA) are widely used due to their objectivity, automation, and quantitative readouts, several comparative studies have shown that IFA may offer similar or superior sensitivity. For instance, Talari et al. [17] reported that IgG-ELISA showed 89% sensitivity compared with IgG-IFA, missing approximately 11% of IFA-positive samples; for IgM, ELISA sensitivity was 78.6%, while IFA detected a higher number of positive samples.

Firouz et al. [18] compared ELISA and chemiluminescent microparticle immunoassay (CMIA) in pregnant women in Iran. They reported an IgG seroprevalence of 52% by ELISA and 56% by CMIA, with no samples testing positive for IgM by either method. The Kappa coefficient (0.841) indicated substantial agreement between the tests. These findings are consistent with our study, which also showed a significant correlation between CMIA and a fluorescence-based method, reinforcing the utility of automated assays employing recombinant antigens in prenatal toxoplasmosis screening. Similarly, Mehrabani et al. [19] compared Dot-ELISA with IFA in 560 children and found a 90% agreement rate, with a Kappa value of 0.73. The authors emphasized the simplicity and rapid turnaround of Dot-ELISA, suggesting it as a viable alternative to IFA in field-based screening contexts.

Naghili et al. [20] evaluated indirect immunofluorescence (IFA), enzyme-linked immunosorbent assay (ELISA), and IgG avidity tests for the detection of anti-*T. gondii* antibodies in 391 pregnant women. The authors observed seropositivity rates of 71.61% by IFA and 68.28% by ELISA, demonstrating a high level of agreement between the methods. In addition, IgG avidity testing helped confirm recent infection in a subset of IgM-positive cases, highlighting the complementary role of different serological approaches in the diagnosis and clinical management of toxoplasmosis during pregnancy.

Nevertheless, MAT is rarely applied in human serological screening, yet it demonstrated strong performance in this study, even slightly outperforming IFA in IgG detection. Its simplicity, low cost, and independence from fluorescence microscopy suggest it may serve as a viable alternative in settings with limited infrastructure. Although MAT is more commonly used in veterinary medicine, particularly in wildlife and livestock diagnostics, it offers important operational advantages [12,21,22]. Notably, it does not require species-specific conjugates or fluorescence microscopy, making it accessible for use in resource-limited laboratories. The differences observed across methods likely reflect not only technical variation in antigen sources and detection thresholds, but also the inherent limitations of interpreting antibody profiles during pregnancy. While CMIA provides operational advantages in busy clinical environments, our findings suggest that conventional methods like IFA and MAT can increase sensitivity and should be considered in confirmatory or complementary roles, particularly in equivocal cases.

These discrepancies highlight the importance of understanding the methodological characteristics and limitations of each serological test. While automated methods like CMIA offer speed and standardization, conventional methods such as IFA and MAT may detect additional cases, especially those with borderline titers. These differences are relevant for clinical decision-making during pregnancy, as false-negative results could delay diagnosis and increase the risk of vertical transmission.

False-negative results in prenatal screening may delay the identification of maternal infection and potentially increase the risk of unrecognized congenital toxoplasmosis. In this context, conventional serological methods such as IFA and MAT may serve as complementary tools in specific diagnostic situations, particularly when clinical suspicion persists despite negative results obtained with automated assays. Nevertheless, the implementation of additional testing strategies should be interpreted with caution, as factors such as laboratory infrastructure, clinical context, and cost-effectiveness must also be considered. These aspects were beyond the scope of the present study.

This study provides one of the first simultaneous comparisons of indirect immunofluorescence assay (IFA), modified agglutination test (MAT), and chemiluminescent microparticle immunoassay (CMIA) for the detection of *T. gondii* antibodies in pregnant women undergoing routine prenatal screening. In this epidemiologically relevant population, IFA and MAT detected a higher number of IgG-positive samples than CMIA, whereas CMIA and IFA showed strong agreement for IgM detection. These findings highlight that conventional serological methods may identify additional IgG-positive cases, particularly in samples with low antibody titers. Within the context of prenatal screening in the Brazilian public healthcare system, where automated assays are widely used, our results suggest that conventional techniques such as IFA and MAT may play an important complementary role. Rather than replacing automated methods, these approaches may contribute to improved diagnostic interpretation and increased accuracy when used in combination during prenatal care.

## 5. Conclusions

IFA and MAT showed greater sensitivity than CMIA in detecting IgG antibodies against *T. gondii* in pregnant women, while CMIA and IFA demonstrated strong agreement for IgM detection. The observed discrepancies highlight the limitations of relying solely on automated methods, reinforcing the value of conventional techniques in improving diagnostic accuracy during prenatal care.

## Figures and Tables

**Table 1:** Contingency table of absolute and relative frequencies of IgG anti-*T. gondii* antibody results in serum samples from pregnant women, according to chemiluminescent microparticle immunoassay (CMIA) and indirect immunofluorescence assay (IFA).

			Total
	IFA (+)	IFA (−)	
**CMIA (+)**	131 (27.93%)	1 (0.21%)	132 (28.14%)
**CMIA (−)**	118 (25.16%)	219 (46.70%)	337 (71.86%)
**Total**	249 (53.09%)	220 (46.91%)	469 (100%)

+: Positive. −: Negative.

**Table 2:** Contingency table of absolute and relative frequencies of IgG anti-*T. gondii* antibody results in serum samples from pregnant women, according to chemiluminescent microparticle immunoassay (CMIA) and modified agglutination test (MAT).

			Total
	MAT (+)	MAT (−)	
**CMIA (+)**	118 (28.36%)	1 (0.24%)	119 (28.61%)
**CMIA (** **−** **)**	95 (22.84%)	202 (48.56%)	297 (71.39%)
**Total**	213 (51.20%)	203 (48.80%)	416 (100%)

+: Positive. −: Negative.

**Table 3:** Contingency table of absolute and relative frequencies of IgM anti-*T. gondii* antibody results in serum samples from pregnant women, according to chemiluminescent microparticle immunoassay (CMIA) and indirect immunofluorescence assay (IFA).

	IFA		Total
	(+)	(−)	
**CMIA**	46 (10.75%)	6 (1.40%)	52 (12.15%)
	4 (0.93%)	372 (86.92%)	376 (87.85%)
**Total**	50 (11.68%)	378 (88.32%)	428 (100%)

+: Positive. −: Negative.

## Data Availability

All data generated or analyzed during this study are included in this article.
